# Race and Ethnicity Influence Survival Outcomes in Women of Caribbean Nativity With Epithelial Ovarian Cancer

**DOI:** 10.3389/fonc.2020.00880

**Published:** 2020-05-29

**Authors:** Matthew Schlumbrecht, Danielle Cerbon, Melissa Castillo, Scott Jordan, Raleigh Butler, Andre Pinto, Sophia George

**Affiliations:** ^1^Division of Gynecologic Oncology, Sylvester Comprehensive Cancer Center, Miami, FL, United States; ^2^Department of Obstetrics and Gynecology, University of Miami Miller School of Medicine, Miami, FL, United States; ^3^Department of Obstetrics and Gynecology, University of the West Indies-Nassau, Nassau, Bahamas; ^4^Department of Pathology, University of Miami Miller School of Medicine, Miami, FL, United States

**Keywords:** ovarian cancer, disparities, Caribbean, Hispanic, race

## Abstract

**Background:** Caribbean immigrants represent one of the largest groups of minorities in the United States (US), yet are understudied. Racial and ethnic disparities among women with ovarian cancer have been reported, but not in immigrant populations. Our objective was to evaluate differences in the clinicopathologic features and survival outcomes of Caribbean-born (CB) immigrants with ovarian cancer, with special focus on the influence of race and ethnicity on these measures.

**Methods:** A review of the institutional cancer registry was performed to identify women with known nativity treated for epithelial ovarian cancer between 2005 and 2017. Sociodemographic, clinical, and outcomes data were collected. Analyses were done using chi-square, Cox proportional hazards models, and the Kaplan-Meier method, with significance set at *p* < 0.05.

**Results:** 529 women were included in the analysis, 248 CB and 281 US-born (USB). CB women were more likely to have residual disease after debulking surgery (31.2 vs. 16.8%, *p* = 0.009) and be treated at a public facility (62.5 vs. 33.5%, *p* < 0.001). Black CB women less frequently received chemotherapy compared to White CB women (55.2 vs. 82.2%, *p* = 0.001). Among all CB women, Hispanic ethnicity was independently associated with improved survival when adjusting for other factors (HR 0.61 [95% CI 0.39–0.95], *p* = 0.03). White Hispanic CB women had a median overall survival (OS) of 59 months while Black, non-Hispanic CB women had a median OS of 24 months (log-rank *p* = 0.04).

**Conclusion:** Among Caribbean-born women with ovarian cancer, Hispanic ethnicity is significantly associated with improved survival outcomes, regardless of race.

## Introduction

Ovarian cancer is the most lethal gynecologic cancer, accounting for a larger number of deaths annually than any other malignancy of the female reproductive tract in the United States (1). Marked racial disparities exist in ovarian cancer outcomes, with Black women demonstrating significantly worse overall survival than White women. In fact, while the 5-years survival for White ovarian cancer patients has improved to nearly 50%, there has been very little change in survival among Black women since the 1970s (2). Access to care, low socioeconomic status, and delays in care due to perceived discrimination have all been suggested as possible contributors to these findings (3, 4); but this may not be the complete story. Recent analyses of The Cancer Genome Atlas (TCGA) determined that tumors from African Americans, across a number of cancer types, demonstrated enriched chromosomal instability compared to tumors from people of European descent (5). In populations of West African descent, distinct genetic loci, not observed in women of European ancestry, have been associated with epithelial ovarian cancer risk (6). These findings suggest that nativity and ancestry are also contributing to disease etiology and outcomes, independent of social determinants.

The Caribbean represents one of the most racially and ethnically diverse regions in the world, with an admixture of peoples from Europe, West Africa, and native Amazonia/Central America (7). Among the populations in the Caribbean are variable contributions from all of these genetic ancestries. Despite the fact that there are known areas of high BRCA mutation prevalence (8), and high rates of gynecologic cancers in some countries like Trinidad and Tobago (9), cancer registries in the Caribbean are poorly developed and both the incidence and outcomes of ovarian cancer are understudied (10). Investigations into other women's cancers, including breast and endometrial, have demonstrated that Black women of Caribbean nativity have unique cancer outcomes, especially when compared to Black women in the United States (11–14). To date, there are no studies examining the effect of race and ethnicity on ovarian cancer among women of Caribbean descent. As the population of Black immigrants to the United States, the majority of which is of Caribbean nativity, has risen by more than 70% over the last 20 years, it is important to understand how the ancestries of these women may influence ovarian cancer outcomes (15). Our objective was to evaluate differences in the clinicopathologic features and survival outcomes of Caribbean-born immigrants with ovarian cancer, with special focus on the influence of race and ethnicity on these measures.

## Methods

After approval from the University of Miami Institutional Review Board (Protocol 2015-1022), all women treated for epithelial ovarian cancer between 2005 and 2017 were identified through the institutional cancer registry. Year of diagnosis was not used as an inclusion criteria, as the date range was large (1964–2017), though the majority of women were diagnosed after 2000. Women for whom country of birth was unavailable were excluded from analysis. Histologies for each patient were reviewed by the principal investigator, with non-epithelial tumors (i.e., germ cell and sex cord stromal cell tumors) and non-invasive tumors (i.e., borderline tumors) also excluded.

Data obtained from the cancer registry included date of diagnosis, age of diagnosis, treatment facility, country of birth, tumor grade and histology, stage at presentation, debulking outcome, insurance status, race and ethnicity, history of alcohol and tobacco use, and receipt of surgery and chemotherapy as components of patient treatment plans. The date of diagnosis was defined as the day of pathologic confirmation of cancer. Age was recorded as a continuous variable. Patient country of birth was dichotomized as either US-born or Caribbean-born, and was self-reported. Race was dichotomized as White or Black (excluding Asian or Unknown Race due to low numbers within the groups). Ethnicity was characterized as either Hispanic or Non-Hispanic. Histology and grade were reviewed by the principal investigator to ensure that the two were consistent, with tumors being segregated into low-grade or high-grade. If the grade and histology were inconsistent (e.g., carcinosarcoma or clear cell carcinoma were incorrectly assigned as low-grade), they were modified in the database. If grade was reported as unknown, but the pathology report indicated a high-grade or low-grade tumor, it was also modified in the database. Additionally, if grade was unclear and tumor specimens were available, they were reviewed by a board-certified Gynecologic Pathologist for diagnostic confirmation (AP). Stage at diagnosis was recorded per AJCC classifications. Alcohol and tobacco use were classified as either never/former or current. Insurance status was classified as private insurance, government or military insurance, or uninsured. Surgery and chemotherapy were dichotomized as given or not given. Debulking status was recorded as optimal (<1 cm gross residual disease) or suboptimal (≥1 cm gross residual disease). Overall survival was defined as time from date of diagnosis to date of death (all-cause).

Statistical analyses were performed using STATA IC 14.2 (StataCorp, College Station, TX). All patients were included in the analyses, even when missing specific data points. Summary statistics were used to describe the patient cohort. Wilcoxon rank-sum was used for continuous variables in non-parametric distributions. Chi-square testing was used to analyze associations between categorical variables. Univariable and multivariable Cox proportional hazards regression, the log-rank test, and the Kaplan-Meier method were utilized to assess survival outcomes. Stepwise backwards multivariable regression analyses included covariates with *p*-values ≤ 0.05 from the univariable models. All tests were two-sided, with significance set at *p* < 0.05.

## Results

During the study interval, 1,210 women received treatment for epithelial ovarian cancer at our institution. Of these, 529 had a known country of birth and were included in the analyses. Demographics of these women are shown in Table 1, and are segregated by nativity. Median age of diagnosis was 59 years for Caribbean-born patients (range 16–87 years) and 57 years for US-born patients (range 17–88 years) (*p* = 0.29). A greater proportion of Caribbean-born women received care at the public facility (62.5 vs. 33.5%, *p* < 0.001) and were Hispanic (74.9 vs. 5%, *p* < 0.001). Though the proportion of women who had surgery as part of their treatment plans was not significantly different between the two groups, the number of Caribbean-born women who had a suboptimal debulking was much greater (31.2 vs. 16.8%, *p* = 0.009). Compared with women born in the US, fewer women born in the Caribbean had any history of smoking (21.3 vs. 31.6%, *p* = 0.01). No differences in the proportion of racial minorities, receipt of chemotherapy, or insurance status were appreciated between the two groups.

**Table 1:** Cohort demographics by nativity.

**Variable**	**Caribbean-born**	**US-born**	***p*-value**
	**(*n* = 248)**	**(*n* = 281)**	
	***N* (%)**	***N* (%)**	
**AGE AT DIAGNOSIS**			
Median (range)	59 (16-87)	57 (17-88)	0.29
**TREATMENT FACILITY**			
Private	93 (37.5)	187 (66.5)	<0.001
Public	155 (62.5)	94 (33.5)	
**HISTOLOGY**			
Serous	161 (79.3)	159 (80.3)	0.80
Non-serous	42 (20.7)	39 (19.7)	
**GRADE**			
Low	49 (29.2)	39 (24.8)	0.38
High	119 (70.8)	118 (75.2)	
**STAGE AT PRESENTATION**			
Stage I–II	41 (22.2)	53 (27.3)	0.25
Stage III–IV	144 (77.8)	141 (72.7)	
**CHEMOTHERAPY**			
No	51 (21.7)	56 (21.4)	0.93
Yes	184 (78.3)	206 (78.6)	
**SURGERY**			
No	42 (17.3)	43 (15.8)	0.65
Yes	201 (82.7)	229 (76.2)	
**DEBULKING STATUS**			
Optimal	77 (68.8)	104 (83.2)	0.009
Suboptimal	35 (31.2)	21 (16.8)	
**ETHNICITY**			
Non-Hispanic	62 (25.1)	266 (95)	<0.001
Hispanic	185 (74.9)	14 (5)	
**RACE**			
White	207 (83.5)	231 (82.2)	0.81
Black	32 (12.9)	38 (13.5)	
Other	9 (3.6)	12 (4.3)	
**SMOKING**			
Never user	174 (78.7)	175 (68.4)	0.01
Former/Current	47 (21.3)	81 (31.6)	
**INSURANCE**			
Private	94 (41.6)	99 (39.4)	0.52
Govt/Military	103 (45.6)	126 (50.2)	
Uninsured	29 (12.8)	25 (10.4)	

Table 2 shows the Cox proportional hazards models for overall survival in the entire cohort. Increasing age at diagnosis (HR 1.04 [95% CI 1.03–1.05], *p* < 0.001), high-grade histology (HR 1.62 [95% CI 1.26–2.10], *p* < 0.001), advanced stage of disease (HR 2.82 [95% CI 2.16–3.69], *p* < 0.001), debulking status (HR 2.02 [95% CI 1.55–2.63], *p* < 0.001), surgical resection of disease (HR 0.28 [95% CI 0.23–0.35], *p* < 0.001), and government/military insurance (HR 1.30 [95% CI 1.09–1.56], *p* = 0.004) were all associated with overall survival in the univariable analysis. Ethnicity, Black race, receipt of chemotherapy, and country of birth had no associations with overall survival. In the multivariable model, only age (HR 1.04 [95% CI 1.02–1.06], *p* < 0.001) and advanced stage of disease (HR 2.15 [95% CI 1.37–3.37], *p* = 0.001) remained independently predictive of survival outcomes.

**Table 2:** Cox proportional hazards modeling of overall survival for the entire cohort.

	**HR**	**CI**	***p*-value**
**FACILITY**			
Private (ref)	–		
Public	1.18	0.99–1.40	0.06
Age at diagnosis	1.04	1.03–1.05	<0.001
**HISTOLOGY**			
Serous (ref)	–		
Non-serous	0.85	0.68–1.08	0.18
**GRADE**			
Low-grade (ref)	–		
High-grade	1.62	1.26–2.10	<0.001
**STAGE**			
Stage I–II (ref)	–		
Stage III–IV	2.82	2.16–3.69	<0.001
**CHEMOTHERAPY**			
No (ref)	–		
Yes	1.02	0.81–1.27	0.89
**SURGERY**			
No (ref)	–		
Yes	0.28	0.23–0.35	<0.001
**SMOKING**			
Never (ref)	–		
Former/Current	1.01	0.84–1.22	0.90
**DEBULKING STATUS**			
Optimal (ref)	–		
Suboptimal	2.02	1.55–2.63	<0.001
**ETHNICITY**			
Non-Hispanic (ref)	–		
Hispanic	0.87	0.74–1.02	0.10
**RACE**			
White (ref)	–		
Non-white	1.62	1.32–1.98	<0.001
**COUNTRY OF BIRTH**			
United States (ref)	–		
Caribbean	1.13	0.90–1.42	0.28
**INSURANCE**			
Private (ref)	–		
Government/Military	1.30	1.09–1.56	0.004
Uninsured	0.83	0.60–1.16	0.29

A sub-analysis of women only born in the Caribbean was next performed, with differences assessed by race. These results are shown in Table 3. The groups were very similar except that Black women received significantly less chemotherapy than White women (55.2 vs. 82.2%, *p* = 0.001), and a larger proportion of Black patients were uninsured (27.6 vs. 10.7%), *p* = 0.001. No differences in histology, grade, or stage were identified between the two groups. Women of reported Hispanic ethnicity represented >75% of the women in each racial cohort. Proportional hazards models for overall survival (Table 4) differed in this group when compared to the larger cohort. While age (HR 1.04 [95% CI 1.02–1.05], *p* < 0.001), advanced stage at presentation (HR 1.97 [95% CI 1.13–3.40], *p* = 0.02), and surgery as part of treatment (HR 0.28 [95% CI 1.18–0.41], *p* < 0.001) remained strongly associated with overall survival in the univariable models, insurance status, debulking status, and tumor grade did not. Additionally, unique to Caribbean-born women was the finding that Hispanic ethnicity was strongly associated with improved overall survival (HR 0.57 [95% CI 0.39–0.83], *p* = 0.003). This finding persisted in the multivariable model, with both Hispanic ethnicity (HR 0.61 [95% CI 0.39–0.95], *p* = 0.03) and receipt of surgery (HR 0.40 [95% CI 0.24–0.67], *p* < 0.001) demonstrating protective effects; advanced age (HR 1.03 [95% CI 1.01–1.05], *p* = 0.001) remained independently associated with worse overall survival.

**Table 3:** Demographics of Caribbean-born patients, by race.

**Variable**	**White**	**Black**	***p*-value**
	***N* (%)**	***N*(%)**	
**AGE AT DIAGNOSIS**			
Median (range)	60 (16-87)	59 (23-87)	0.67
**TREATMENT FACILITY**			
Private	77 (37.2)	10 (31.3)	0.52
Public	130 (62.8)	22 (68.7)	
**HISTOLOGY**			
Serous	132 (77.6)	22 (88)	0.24
Non-serous	38 (22.7)	3 (12)	
**GRADE**			
Low	39 (27.3)	7 (35)	0.47
High	104 (72.7)	13 (65)	
**STAGE AT PRESENTATION**			
Stage I–II	37 (23.3)	2 (11.1)	0.24
Stage III–IV	122 (76.7)	16 (88.9)	
**CHEMOTHERAPY**			
No	35 (17.8)	13 (44.8)	0.001
Yes	162 (82.2)	16 (55.2)	
**SURGERY**			
No	35 (17.2)	6 (20)	0.70
Yes	169 (82.8)	24 (80)	
**DEBULKING STATUS**			
Optimal	65 (68.4)	8 (72.7)	0.77
Suboptimal	30 (31.6)	3 (27.3)	
**ETHNICITY**			
Non-Hispanic	49 (23.8)	8 (25.0)	0.88
Hispanic	157 (76.2)	24 (75.0)	
**SMOKING**			
Never user	145 (78.8)	23 (82.1)	0.69
Former/Current	39 (21.2)	5 (17.9)	
**INSURANCE**			
Private	84 (44.4)	8 (27.6)	0.03
Govt/Military	85 (44.9)	13 (44.8)	
Uninsured	20 (10.7)	8 (27.6)	

**Table 4:** Cox proportional hazards modeling of overall survival among Caribbean-born women with ovarian cancer.

	**HR**	**CI**	***p*-value**
**FACILITY**			
Private (ref)	–		
Public	1.13	0.80–1.59	0.49
Age at diagnosis	1.04	1.02–1.05	<0.001
**HISTOLOGY**			
Serous (ref)	–		
Non-serous	0.71	0.43–1.17	0.19
**GRADE**			
Low-grade (ref)	–		
High-grade	1.48	0.90–2.45	0.12
**STAGE**			
Stage I–II (ref)	–		
Stage III–IV	1.97	1.13–3.40	0.02
**CHEMOTHERAPY**			
No (ref)	–		
Yes	1.09	0.68–1.74	0.72
**SURGERY**			
No (ref)	–		
Yes	0.28	0.18–0.41	<0.001
**SMOKING**			
Never (ref)	–		
Former/Current	0.93	0.60–1.45	0.76
**DEBULKING STATUS**			
Optimal (ref)	–		
Suboptimal	1.52	0.85–2.71	0.16
**ETHNICITY**			
Non-Hispanic (ref)	–		
Hispanic	0.57	0.39–0.83	0.003
**RACE**			
White (ref)	–		
Black	1.19	0.70–2.05	0.52
**INSURANCE**			
Private (ref)	–		
Government/Military	1.40	0.88–2.25	0.15
Uninsured	0.75	0.37–1.52	0.42

**Figure 1:**
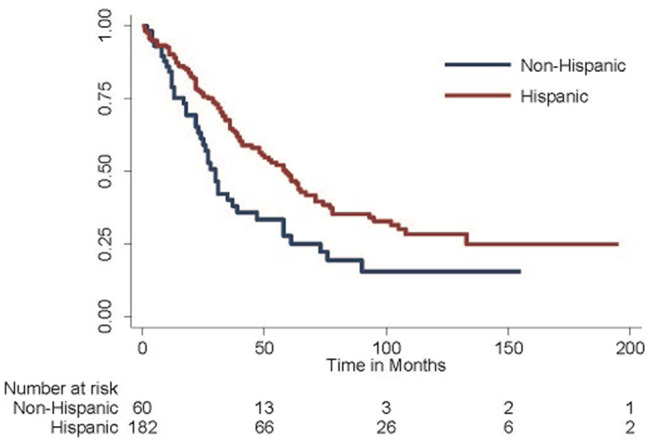
Overall survival among Hispanic-Caribbean. Among women born in the Caribbean with epithelial ovarian cancer, those of Hispanic ethnicity do better than those of non-Hispanic ethnicity (median overall survival 59 vs. 30 months, log-rank *p* = 0.002).

**Figure 2:**
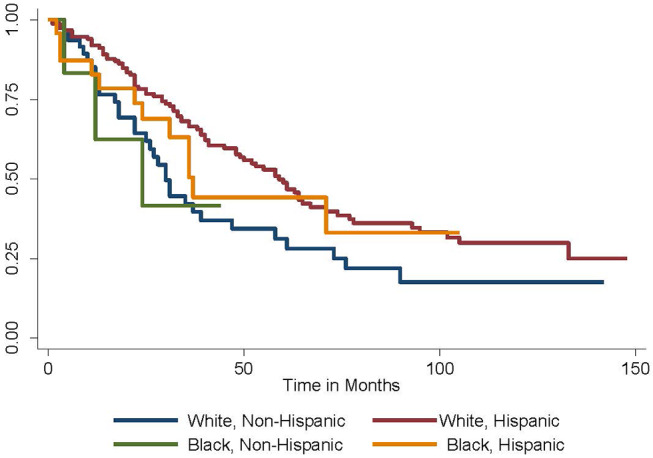
Overall survival by race and ethnicity among Caribbean women with ovarian cancer. Hispanic ethnicity is protective, while Black race is associated with worse survival (log-rank *p* = 0.04).

Given these findings, the Kaplan-Meier method was employed to generate survival curves by race and ethnicity within the Caribbean-born cohort (Figures 1, 2). Hispanic ethnicity was associated with a median improvement in overall survival of 29 months (log-rank *p* = 0.002). When both race and ethnicity were considered together, Hispanic race remained protective regardless of race (log-rank *p* = 0.04). White Hispanic women had a median overall survival of 59 months compared to the 30 month median overall survival for non-Hispanic White women. Hispanic ethnicity was also protective among women of Black race, with non-Hispanic Blacks demonstrating a median overall survival of 24 months and Hispanic Blacks a median overall survival of 37 months.

## Discussion

Disparities in ovarian cancer care by race and ethnicity have been previously reported, however in this investigation we provide specific data about an understudied group of Caribbean-born women who received treatment in the United States. Several studies have demonstrated that survival among Black women with ovarian cancer is worse than other races. Stewart et al. reported on an large cohort of more than 172,000 women, and demonstrated that independent of time period, stage at presentation, and geography, Black women had at least 10% lower 5-years survival than White women (16). Such trends have been demonstrated across all histologic subtypes, as well (17). A recent investigation using the Florida Cancer Data System, and thus potentially relevant to our current population of study, also showed that Black women have significantly worse overall survival, even when adjusting for demographic factors, tumor characteristics, and treatment types (18).

Women who were Caribbean-born were nearly twice as likely to undergo a suboptimal debulking, whereby significant residual disease is left behind at the time of surgery. As all patients were treated in the same center under the same care team, this is likely not reflective of practice variations and may be more indicative of tumor aggressiveness leading to inability to surgically resect. The appearance of more aggressive tumors in populations of the Caribbean has been reported in a number of disease sites. Tonon et al. reported on significantly higher mortality among prostate cancer patients in Afro-Caribbean men compared to those of European ancestry, suggesting that observed mutations in genes along the DNA damage repair pathway may be contributing to differences in disease phenotype (19). It has been shown that Caribbean-born women with breast cancer have greater proportions of triple negative breast cancers and higher-stage disease at presentation (14). Among Black women of Caribbean nativity with endometrial cancer, there is a higher proportion of high-grade histologies and advanced stages at presentation (12). One factor which may be potentially confounding our data is the heterogeneity within the stages of ovarian cancer. Stage III disease, for example, may be limited to the omentum, or may involve bulky, disseminated intra-abdominal disease. We are unable to determine this degree of subtlety using a database. If this were the case, however, it would still speak to some variation in the biology of this disease between the two groups, and suggest that a molecular driver of invasiveness and severity of dissemination should be considered as an etiology for this observation.

Chemotherapy utilization was significantly lower in the population of Afro-Caribbean women compared to White-Caribbean women (55.2 vs. 82.2%, *p* = 0.001). Both physiologic and sociocultural influences may be contributing to this finding. It has been well-described that Black women have lower white blood cell (WBC) counts compared to women of European ancestry (20). As WBC are the cells that mount an immune response against infection, adequacy of counts is crucial for both initiation and continuation of chemotherapy. Among Black patients with breast cancer, this phenomenon of low WBC counts has been well-described, leading to delays in treatment, and failure to dose-escalate (21, 22). Whether or not this translates into survival disadvantages in ovarian cancer has yet to be elucidated, but may be a reason for fewer Black women receiving treatment in this cohort. From a behavioral perspective, a number of factors may influence the decision to proceed with chemotherapy. Employment status, poor body image, and low emotional support have all been cited as factors contributing to treatment adherence among Black women (23, 24). In fact, Black women with ovarian cancer have been shown to have a >50% risk of non-guideline adherent care compared with white women (25). Among African and Afro-Caribbean men undergoing treatment for prostate cancer, misunderstanding about treatment modalities and side effects, as well as lack of trusting relationships with health professionals, is associated with willingness to undergo treatment (26). Delays in ovarian cancer care also have been strongly associated with religiosity and inaccurate cultural/folk beliefs about cancer treatment among African American women (27). Such findings all suggest that concerted patient education efforts are necessary, particularly in groups at risk for inadequate treatment completion.

Perhaps the most striking finding of our study is the significant positive influence Hispanic ethnicity had on survival, for both White and Black races, among women born in the Caribbean. In an independent large cohort of ovarian cancer patients in Florida, Hispanic ethnicity was associated with overall survival advantages, but it did not reach statistical significance (18). In a breast cancer cohort, Hispanic ethnicity did result in improved overall survival (14). Utilizing large, publicly available national databases, other authors have demonstrated that Hispanic ethnicity has no impact on ovarian cancer survival (17, 28). Within our study, Hispanic ethnicity only appeared to be beneficial among women born in the Caribbean, with a robust 39% reduction in the risk of death. The reason for this finding is unclear. The Hispanic mortality paradox, whereby despite socioeconomic disadvantages Hispanic patients have improved survival, has been validated in several studies (29, 30), but none specific to ovarian cancer. The effect of Hispanic ethnicity may not even be uniform. An evaluation of SEER data completed by Chen et al. examining a period between 1992 and 2013 revealed that there was significant variation in survival outcomes by Hispanic sub-ethnicity, with Puerto Ricans and Cubans faring much better than Mexicans and Other Hispanics (31). Evaluation of sub-populations of Hispanics is important as studies of other gynecologic malignancies have also demonstrated variable risk between these groups (32). The distinct, independent survival benefit of Hispanic ethnicity among Caribbean-born women with ovarian cancer may be related to sociocultural or behavioral effects, or reflect phenotypic displays of complex genetic admixture. Further study to explain this phenomenon is warranted.

Our study has several limitations. Like any retrospective study, inaccuracy of data due to poor or incomplete reporting may have biased the results. As a single-institution report, our findings may be applicable only to our population and have limited applicability elsewhere. However, given the unique geographic proximity of South Florida to the Caribbean, and considering the large numbers of immigrants who reside in our state, a study, such as this may not be achievable elsewhere. Validation of these data with larger datasets, either from state or international registries, may provide further information to corroborate these findings. Additionally, as these patients were treated by the same group of physicians, variations in practice patterns which could otherwise introduce bias into decision-making regarding surgery and how aggressive the attempts at debulking were, as well as utilization of chemotherapy as adjunct treatment, are likely minimized, even as national treatment algorithms evolved over the study period. Genetic status, which is known to significantly influence response to chemotherapy and survival outcomes, was not available for most of the cases, as routine genetic testing was not performed on all patients until toward the end of the study interval as per National Comprehensive Cancer Network (NCCN) Guidelines (33). The number of patients for whom nativity was available was less than half of the overall cohort, which limits our power to potentially detect more subtle differences in a robust manner, or to further evaluate patterns of disease and survival by specific countries of birth. As race/ethnicity are not monolithic, and prior studies have demonstrated variations within sub-populations of larger racial/ethnic groups, more granular investigations and risk stratification are crucial. Within our cohort, of the 1,210 women for consideration, only 43.7% had reported nativity, potentially biasing our findings. This lack of data represents a limitation of retrospective investigations, and in this case, capture of relevant patient demographics. As a sanctuary city, hesitation of immigrants to report nativity or undocumented status may have occurred, and is unfortunately a common concern in populations similar to the one investigated (34). Despite this, our data are compelling in that they demonstrate significant associations by race and ethnicity in an understudied population, and are suggestive of additional necessary investigations into the molecular, genetic, and bio-behavioral mediators of disease pathogenesis and treatment response.

## Conclusion

Ovarian cancer remains the leading cause of gynecologic cancer death, a status which has not changed over the last decade. In this investigation we have demonstrated that both race and ethnicity appear to influence overall survival among Caribbean-born women with epithelial ovarian cancer. While the importance of developing novel therapeutics cannot be understated, a deeper understanding of the factors contributing to variations in personal risk and outcomes is needed. The hereditary basis of disease, pharmacogenomics, and sociocultural approaches to treatment all require exploration in an attempt to truly personalize care delivery. Such research should also seek to translate into meaningful strategies for prevention and advocacy, as well as foster international collaborations with our Caribbean neighbors to address this morbid disease on a more global scale.

## Data Availability Statement

The raw data supporting the conclusions of this article will be made available by the authors, without undue reservation.

## Ethics Statement

The studies involving human participants were reviewed and approved by University of Miami Institutional Review Board. Written informed consent for participation was not required for this study in accordance with the national legislation and the institutional requirements.

## Author Contributions

MS and SG developed the research concept, design, and wrote the manuscript. MS conducted the data analysis. DC, MC, and SJ abstracted the data and did data cleaning. RB contributed to the manuscript. AP reviewed the necessary pathology slides and contributed to the manuscript. Final submission approved by all authors.

## Conflict of Interest

The authors declare that the research was conducted in the absence of any commercial or financial relationships that could be construed as a potential conflict of interest.
